# Immunotherapy Combined With Radiation Therapy for Genitourinary Malignancies

**DOI:** 10.3389/fonc.2021.663852

**Published:** 2021-05-10

**Authors:** Jacob Ukleja, Erika Kusaka, David T. Miyamoto

**Affiliations:** ^1^ Department of Radiation Oncology, Massachusetts General Hospital, Harvard Medical School, Boston, MA, United States; ^2^ Massachusetts General Hospital Cancer Center, Charlestown, MA, United States

**Keywords:** immunotherapy, radiation therapy, renal cancer, bladder cancer, prostate cancer, genitourinary cancer (GU cancer), radiotherapy, immune checkpoint inhibitor

## Abstract

Immunotherapy drugs have recently been approved by the Food and Drug Administration for the treatment of several genitourinary malignancies, including bladder cancer, renal cancer, and prostate cancer. Preclinical data and early clinical trial results suggest that immune checkpoint inhibitors can act synergistically with radiation therapy to enhance tumor cell killing at local irradiated sites and in some cases at distant sites through an abscopal effect. Because radiation therapy is commonly used in the treatment of genitourinary malignancies, there is great interest in testing the combination of immunotherapy with radiation therapy in these cancers to further improve treatment efficacy. In this review, we discuss the current evidence and biological rationale for combining immunotherapy with radiation therapy, as well as emerging data from ongoing and planned clinical trials testing the efficacy and tolerability of this combination in the treatment of genitourinary malignancies. We also outline outstanding questions regarding sequencing, dose fractionation, and biomarkers that remain to be addressed for the optimal delivery of this promising treatment approach.

## Introduction

Prostate, bladder, and kidney/renal pelvis cancers rank fourth, seventh, and eighth, respectively, in estimated cancer-related deaths in the United States in 2020 (1). Radiation therapy is a well-established treatment modality for genitourinary malignancies, with clinical utility in the definitive, adjuvant, and palliative settings. In localized prostate cancer, for example, radiation therapy is a curative treatment option with survival outcomes that have been shown to be equivalent to those of radical prostatectomy (2). In bladder cancer, radiation is a critical part of bladder-preserving trimodality therapy, which has comparable outcomes to radical cystectomy in well-selected patients (3). Renal cell carcinoma has traditionally been considered relatively radioresistant, but recent advances in radiation delivery and image guidance technologies have led to the development of stereotactic body radiotherapy (SBRT), which enables the focal and conformal delivery of ablative radiation doses sufficient for the definitive treatment of primary renal cancer (4). In patients with metastatic cancer, palliative radiotherapy is frequently used to alleviate pain from bone metastases. In addition, emerging data suggests that in oligometastatic cancers with five or fewer metastatic lesions, the aggressive use of SBRT to ablate all sites of metastatic disease can lead to improved clinical outcomes (5, 6).

The last several years have also seen the rapid availability of immunotherapy drugs that increase overall survival in patients with a variety of cancers, including genitourinary malignancies (7). Immunotherapy utilizes the patient’s immune system to induce tumor cell killing and can be either active or passive in nature. Active immunotherapy directly targets tumor cells and includes antibody therapy and chimeric antigen receptor T-cell therapy. In contrast, passive immunotherapy enhances the ability of the immune system to eradicate tumor cells and includes checkpoint inhibitors and cytokines. Among these approaches, immune checkpoint inhibitors have shown some of the most promising clinical activity to date. Currently available checkpoint inhibitors target two immune checkpoints: PD-1/PD-L1, which modulates T-cell activity resulting in immune response inhibition (8), and CTLA-4, an immunoglobulin expressed by activated T cells that downregulates immune response (9). FDA-approved therapies that target these immune checkpoints include atezolizumab, durvalumab, pembrolizumab, nivolumab (PD-1/PD-L1), and ipilimumab (CTLA-4), among others (10, 11).

Recent data suggest that radiotherapy and immunotherapy may act synergistically, and there has been mounting excitement about the possibility of combining these modalities to further improve outcomes in patients with genitourinary cancers. In this review, we discuss the pre-clinical mechanistic rationale for combining radiotherapy with immunotherapy, as well as emerging data from ongoing and planned clinical trials testing the efficacy and tolerability of this combination in genitourinary malignancies.

## Biological Rationale for Combining Radiotherapy and Immunotherapy

### Radiotherapy Can Augment Immunotherapy

**Figure 1:**
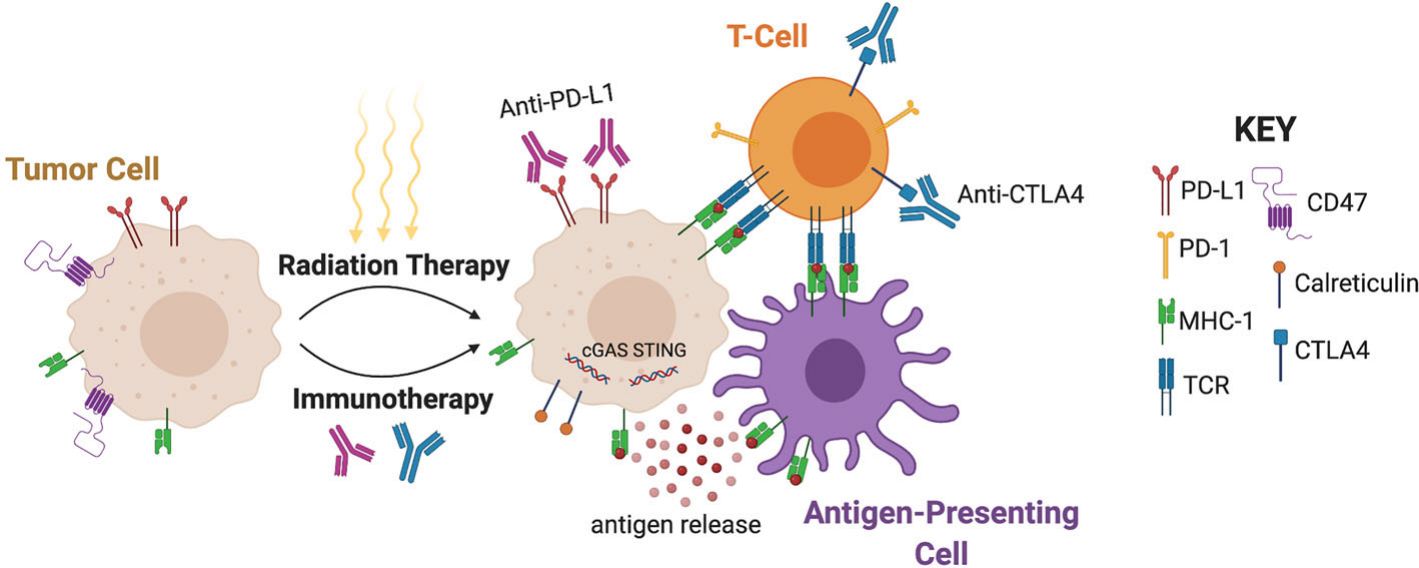
Mechanisms underlying synergy of radiotherapy and immunotherapy. Radiation promotes the ability of antigen-presenting cells to present tumor antigens to naive T cells through antigen release, stimulation of calreticulin, and downregulation of CD47. MHC-1 expression and the subsequent antigen presentation leads to interaction with T-Cell Receptors (TCR). Moderate doses of radiation also activate a type I interferon response through the sensing of cytoplasmic DNA *via* cGAS-STING. Radiation can upregulate PD-L1 and CTLA-4, and therefore immunotherapy can augment radiation efficacy by targeting these pathways. (Created with BioRender.com).

Several lines of evidence suggest that radiation can stimulate the tumor immune microenvironment, a concept that underlies a key rationale for combining radiotherapy with immunotherapy (12). In many cancers, the immune microenvironment becomes altered from a state of immune recognition/antagonism towards a state of immune escape, where the immune system becomes incapable of combatting the tumor (13). Biological changes commonly associated with immune escape include reduced MHC-class 1 expression, upregulated inhibitory ligands and cytokines, and increased numbers of myeloid-derived suppressor cells (14). Although the primary mechanism by which radiation causes local cell death is through the induction of DNA double-strand breaks (15), radiation has been shown to be immunogenic through the direct and indirect activation of innate and adaptive immune response **(** **Figure 1** **)** (16). Local cell death caused by radiation instigates the direct release of tumor antigens and promotes the priming and activation of cytotoxic T cells. In addition, radiation can promote the ability of antigen-presenting cells to present tumor antigens to naive T cells through the stimulation of calreticulin, a calcium-binding protein that promotes phagocytosis (17, 18). Conversely, radiation has also been found to downregulate the presence of CD47, a protein that signals down-regulation of phagocytosis (19). High radiation doses have been shown to increase MHC-1 expression, increasing the likelihood of tumor-specific peptide presentation by antigen-presenting cells to naïve T cells (20). This phenotype, in conjunction with increased expression of death receptors such as Fas, facilitates the immune system’s ability to kill tumor cells by enhancing the visibility of the tumor to cytotoxic T cells (21, 22). Moderate doses of radiation have also been shown to activate a type I interferon response in tumor cells through the sensing of cytoplasmic DNA derived from tumor micronuclei *via* the cGAS-STING pathway (23–26). Through these different processes, radiation therapy ultimately creates a proinflammatory microenvironment that instigates immune activation in a manner that may be synergistic with immunotherapy.

### Immunotherapy May Augment Radiotherapy

Not all tumors will respond to radiation, despite administration of definitive doses. Although the reason for radioresistance remains unclear, one hypothesis is that immune-mediated mechanisms may be involved (27). It is important to note that although radiation can be immunogenic, it can also be immune-suppressive. Radiation can directly kill immune cells in or near the tumor through DNA double strand breaks and apoptotic cell death, which in turn may negatively impact T cells in peripheral circulation (28). For example, a retrospective study of prostate cancer patients treated with (N=36) or without (N=95) pelvic nodal irradiation demonstrated a higher risk of radiation-related lymphopenia with pelvic nodal irradiation (29). Indirectly, while activation of type 1 interferon through cGAS-STING induces recruitment of effector T cells and antigen presenting cells (30), it can also upregulate transforming growth factor β (TGF-β), which triggers an immune-suppressive environment (31–33). Radiation can also drive the recruitment of myeloid-derived suppressor cells (MDSCs) (34), which serve as critical mediators of immunosuppression and inhibit effector T cells as well as induce Tregs (35). Increased infiltration of Tregs into the tumor microenvironment through radiation can downregulate the immune response (36). As a result, radiation’s impact on MDSCs and T cells may promote tumor growth, local invasion, and subsequent metastases (37). Thus, therapies that counteract this effect by augmenting T-cell function may lead to improved control of the tumor (38). Radiation can also alter the balance of key immune checkpoint pathways including PD-L1 and CTLA-4. Radiation temporarily upregulates PD-L1 in mice with bladder cancer (39). The binding of the PD-L1 protein to the inhibitory checkpoint molecule PD-1 reduces the proliferation of antigen-specific T cells in lymph nodes (40). Similarly, radiation can upregulate the CTLA-4 receptor in T cells, leading to a downregulated immune response (41, 42). Thus, an important rationale for incorporating immunotherapy into radiotherapy regimens is to augment the efficacy of radiation by selectively targeting these immune suppressive effects.

### Radiotherapy and Immunotherapy Are Synergistic

Compared to other cancer treatments, tumor response to immunotherapy is often slower and may result in transient increases in tumor burden, even in patients who have an effective immune response (43). Radiotherapy could potentially greatly reduce the growth of such tumors, thus enabling patients to respond to the immunotherapy for longer periods of time (44). In a similar vein, radiation can be used to prime the tumor for immunotherapy by increasing the susceptibility of tumor cells to immune-mediated treatment (45). Moreover, combining immune modulating agents and radiation may induce protective immunologic memory, which could prevent disease recurrence. Finally, reports in the literature suggest that combining immune checkpoint inhibitors and radiotherapy may result in increased frequency of the “abscopal effect,” the immunogenic cell killing of untreated distant tumors (46). Although the potential mechanism for the abscopal effect may include radiation-induced stimulation of systemic recognition of tumor-related antigens, the overall rarity of clinical cases necessitates further investigation (46, 47).

## Clinical Evidence for Combining Radiotherapy and Immunotherapy

### Non-Genitourinary Cancers

Several clinical studies have demonstrated a benefit for the combination of radiotherapy and immunotherapy in non-genitourinary cancers, as reviewed comprehensively elsewhere (44). For example, in lung cancer, the PACIFIC trial enrolled 709 non-small cell lung cancer (NSCLC) patients previously treated with platinum-based chemoradiation and randomized them in a 2:1 ratio to receive either adjuvant durvalumab or placebo. Treatment with durvalumab resulted in an increase in median progression-free survival and 2-year overall survival (66.3% *vs* 55.6%, P=0.005) (48, 49). In a secondary analysis of the KEYNOTE-001 phase 1 trial of pembrolizumab in NSCLC, patients who had previously received radiation therapy prior to receiving pembrolizumab experienced an increased median progression-free survival and overall survival compared to patients without previous radiotherapy (50). In the PEMBRO-RT Phase 2 randomized trial, 76 NSCLC patients received either pembrolizumab and SRBT (3 x 8 Gy within 7 days prior to the first cycle) or pembrolizumab alone. The study found that pembrolizumab preceded by SBRT resulted in a doubling of the overall response rate at 12 weeks (36% *vs* 18%, P=0.07) without any significant increase in toxicity, although this did not meet the prespecified endpoint for meaningful clinical benefit (51). Interestingly, subgroup analyses showed the largest benefit from the addition of radiation in patients with PD-L1 negative tumors.

### Prostate Cancer

Although numerous clinical trials are investigating the combination of radiotherapy and immunotherapy in genitourinary cancers **(** **Table 1** **)**, only a few randomized trials have been published to date with mature results. In prostate cancer, a multicenter phase 3 trial investigated the use of ipilimumab *vs.* placebo after bone-directed radiotherapy (8 Gy x 1 fraction) in 799 men with metastatic castration-resistant prostate cancer that progressed after docetaxel (63). Ipilimumab therapy was associated with a trend towards increased overall survival that was not statistically significant (P=0.053). However, subgroup analyses suggested that patients with favorable prognostic features such as the absence of visceral metastasis or anemia and normal alkaline phosphatase did have a significant improvement in survival with the addition of ipilimumab (63). In a phase 2 trial, 49 patients with oligometastatic hormone-sensitive prostate cancer were randomized to receive either the autologous cellular immunotherapy sipuleucel-T preceded by radiotherapy (30 Gy to a single metastatic site) or sipuleucel-T alone (52). Median progression-free survival was higher with the addition of radiotherapy (3.65 *vs.* 2.46 months, P=0.06), but this was not statistically significant. Overall, radiotherapy did not significantly enhance the humoral and cellular responses associated with sipuleucel-T.

**Table 1:** Active Phase II and III clinical trials combining immunotherapy with radiation therapy in genitourinary cancers.

Cancer	Study	Eligibility	Design	Intervention	Planned Enrollment	Ref
Prostate	NCT01436968	Localized PC	Phase III	RT + valacyclovir ± AdV-tK ± Aglatimagene besadenovec (CAN-2409)	711	–
Prostate	NCT02107430	Localized High-Risk PC	Phase II	RT ± Dendritic Cells (DCVAC/PCa)	62*	–
Prostate	NCT01807065	mCRPC	Phase II	Sipuleucel-T ± RT	51*	(52)
Prostate	NCT01818986	mCRPC	Phase II	SBRT + Sipuleucel-T	20*	–
Prostate	NCT03007732	Newly Diagnosed Hormone-Naive Oligometastatic PC	Phase II	SBRT + ADT + Pembrolizumab ± TLR9 agonist (SD-101)	42	–
Prostate	NCT03795207	Oligometastatic Recurrent Hormone Sensitive PC	Phase II	SBRT ± Durvalumab	96	(53)
Urothelial	NCT02662062	MIBC	Phase II	RT + cisplatin + Pembrolizumab	30	(54)
Urothelial	NCT03171025	Localized MIBC	Phase II	Chemoradiation with Adjuvant Nivolumab	28	(55)
Urothelial	NCT02621151	MIBC	Phase II	RT + Gemcitabine + Pembrolizumab	54*	–
Urothelial	NCT03421652	Locally Advanced UC Ineligible for Chemotherapy	Phase II	RT + Nivolumab	34	–
Urothelial	NCT03775265	Localized MIBC	Phase III	Chemoradiation ± Atezolizumab	475	(56)
Urothelial	NCT03950362	BCG Unresponsive NMIBC	Phase II	RT + Avelumab	67	–
Urothelial	NCT04543110	MIBC	Phase II	RT + Durvalumab	25	–
Urothelial	NCT03747419	MIBC	Phase II	RT + Avelumab	24	–
Urothelial	NCT03702179	MIBC	Phase II	RT + Durvalumab + Tremelimumab	32	(57)
Urothelial	NCT04216290	Node-positive Bladder Cancer	Phase II	Chemotherapy + RT ± Durvalumab	114	–
Urothelial	NCT03915678	anti-PD-1/L1 refractory Bladder Cancer ‡	Phase II	RT + Atezolizumab + BDB001	247	–
Urothelial	NCT03529890	Locally Advanced UC	Phase II	Neoadjuvant RT + Nivolumab	33	–
Urothelial	NCT03115801	Metastatic UC	Phase II	Atezolizumab or Pembrolizumab ± RT	112	–
Urothelial	NCT03511391	UC ‡	Phase II	(Pembrolizumab or Nivolumab or Atezolizumab) ± SBRT	99*	–
Renal	NCT01896271	Metastatic ccRCC	Phase II	SBRT + HD IL-2	26	(58)
Renal	NCT03065179	Metastatic ccRCC	Phase II	SBRT + Nivolumab + Ipilimumab	29*	(59)
Renal	NCT02306954	Metastatic RCC	Phase II	HD IL-2 ± SBRT	84	–
Renal	NCT02781506	Metastatic ccRCC	Phase II	SBRT + Nivolumab	7*	–
Renal	NCT01884961	Metastatic ccRCC ‡	Phase II	SBRT + HD IL-2	35	(60)
Renal	NCT03050060	Metastatic ccRCC ‡	Phase II	hypofractionated RT + Nelfinavir + (Pembrolizumab or Nivolumab or Atezolizumab)	120	–
Renal	NCT02599779	Metastatic RCC	Phase II	SBRT + Pembrolizumab	35	–
Renal	NCT03115801	Metastatic RCC	Phase II	Nivolumab ± RT	112	–
Renal	NCT03469713	Metastatic RCC	Phase II	SBRT + Nivolumab	69*	(61)
Renal	NCT03511391	RCC ‡	Phase II	Nivolumab ± SBRT	99*	–
Renal	NCT02992912	Metastatic RCC ‡	Phase II	SBRT + Atezolizumab	187	–
Renal	NCT04090710	Metastatic RCC	Phase II	Ipilimumab/Nivolumab± SBRT	78	(62)

BCG, Bacillus Calmette-Guerin; ccRCC, clear cell renal cell carcinoma; HD IL-2, high dose IL-2; mCRPC, metastatic castration-resistant prostate cancer; MIBC, muscle-invasive bladder cancer; PC, prostate cancer; RT, radiation therapy; RCC, renal cell carcinoma; SBRT, stereotactic body radiation therapy; UC, urothelial carcinoma.

*Actual completed enrollment.

^‡^For trials enrolling multiple cancer types, details are provided only for the GU cancer arms.

Although these clinical trials have not demonstrated a definite benefit for the addition of radiotherapy to immunotherapy, results from additional ongoing clinical trials in prostate cancer are pending, including those testing PD-1/PD-L1 checkpoint inhibitors in combination with radiotherapy (**Table 1**). For example, in an ongoing phase 2 study (NCT03795207), 96 oligometastatic prostate cancer patients are randomized to either SBRT with durvalumab or SBRT alone in a 2:1 manner. Durvalumab (1500 mg/cycle) is administered one month prior to SBRT (3 x 9 Gy or 3 x 11 Gy) and continued until progression with a maximum of 12 months. The primary endpoint of the trial is 2-year progression-free survival (53).

### Kidney Cancer

Immune checkpoint inhibition has become a standard of care treatment for patients with metastatic renal cell carcinoma (RCC) (64, 65). Multiple clinical trials are currently evaluating whether the addition of radiotherapy to immunotherapy will further improve outcomes in this disease (**Table 1**). Early results have been presented for the RADVAX RCC single arm phase 2 trial (NCT03065179), in which 25 metastatic RCC patients received nivolumab and ipilimumab (N/I) with SBRT (50 Gy in 5 fractions) between the first and second doses of N/I (59). Partial responses were observed in 14/25 patients, for an objective response rate of 56%, which is higher than the expected response rate of 40%. The regimen was noted to have acceptable safety, although 10 (40%) patients required prednisone for immune-related adverse events. These results are encouraging for further investigation, although the study is limited by its small sample size and single-site design.

Preliminary results of the NIVES single arm phase 2 multicenter study (NCT03469713) have been presented recently, in which patients with metastatic RCC that progressed on up to two prior systemic therapies were treated with nivolumab for 6 months, in combination with SBRT (10 Gy x 3 fractions) to one metastatic lesion given 7 days after initiation of nivolumab (61). At a median follow-up of 15 months, the objective response rate was 17.4% (12/68 patients). Although tolerability was acceptable [most frequent grade 3/4 toxicities were diarrhea (5.8%), elevated amylase/lipase (4.3%), and fatigue (4.3%)], the study did not meet its primary endpoint of improving response rate to 40%.

Overall, the available results for combining immunotherapy with radiotherapy are mixed in RCC. Additional data from ongoing clinical trials are anticipated to clarify whether changing the timing or target site of SBRT will further improve outcomes. For example, to test the strategy of targeting the primary kidney lesion with SBRT rather than targeting metastases in this context, the CYTOSHRINK phase 2 trial (NCT04090710) will randomize up to 78 untreated advanced RCC patients to receive ipilimumab/nivolumab plus SBRT to the primary lesion (30-40 Gy in 5 fractions) between cycles 1 and 2, or ipilimumab/nivolumab alone (62).

### Bladder Cancer

Although muscle-invasive bladder cancer has historically been treated with radical cystectomy, bladder-preserving trimodality therapy consisting of transurethral tumor resection, radiotherapy, and chemotherapy is now considered a standard treatment option according to consensus clinical guidelines (66, 67). Several clinical trials are examining the potential role of adding immunotherapy to further improve outcomes of these patients (**Table 1**). A phase Ib study (NCT02891161) demonstrated the safety of combining the anti-PD-L1 checkpoint inhibitor durvalumab with radiation therapy to the bladder (64.8 Gy in 36 fractions) in 6 patients with locally advanced bladder cancer, with no patients experienced dose limiting toxicity (68). A follow-up randomized phase 2 study (ECOG-ACRIN/NRG 8185; NCT04216290) is examining the addition of durvalumab to chemoradiation therapy in patients with clinically node-positive (N1-2) muscle-invasive bladder cancer. A large cooperative group randomized phase 3 study (SWOG/NRG 1806; NCT03775265) with a planned accrual of 475 patients is investigating the addition of the anti-PD-L1 inhibitor atezolizumab to chemoradiation in patients with localized muscle-invasive bladder cancer. Safety data from the first 73 patients of this study were recently presented, showing no grade 3 or higher immune-related adverse events to date (56). Another study is exploring the potential of this strategy for the management of non-muscle invasive bladder cancer, using the combination of radiotherapy with Bacillus Calmette-Guerin (BCG) and durvalumab (ADAPT-bladder; NCT03317158).

## Considerations Surrounding Combining Radiotherapy and Immunotherapy

### Sequencing

The optimal timing and sequencing of radiotherapy and immunotherapy for maximum efficacy of combination therapy remain unknown, although these may vary depending on tumor histology and type of immunotherapy (13). Interestingly, in a post-hoc analysis of the PACIFIC trial, patients who received durvalumab within 14 days after completing chemoradiation had better progression free survival than those who received durvalumab after 14 days, suggesting that immunotherapy should be started soon after radiation (69). Similarly, in a retrospective review of 758 patients with a range of cancer diagnoses who received radiotherapy and immunotherapy (either anti-CTLA-4 or anti-PD-1/PD-L1), patients who received concurrent therapy had better overall survival. Moreover, of those who received concurrent therapy, patients who received induction immunotherapy starting more than 30 days before radiation had improved overall survival compared to those who started less than 30 days before radiation (70). These studies suggest that careful consideration needs to be given to timing and sequencing of radiotherapy and immunotherapy in the design of clinical trials.

### Dose and Fractionation

The optimal radiation dosing and fractionation strategy to maximize immunogenicity remains controversial. Most lines of evidence suggest that higher doses of radiation (>6-8 Gy per fraction) are more immunogenic than typical doses used in conventional fractionation (1.8-2 Gy per day) (71–73). Moderately high doses of 8-12 Gy seem to optimally activate the type I interferon response *via* cGAS/STING, while very high doses (20-30 Gy in 1 fraction) result in a decline in radiation-induced STING activation, in part due to negative feedback inhibition by Trex1 exonuclease which reduces accumulation of cytoplasmic DNA (24). Ultimately, the various fractionation schemes incorporated into ongoing clinical trials will yield insights into the optimal radiation dosing and fractionation needed for the effective combination with immunotherapy.

### Biomarkers of Efficacy and Toxicity

The efficacy of immunotherapy varies greatly across patients and cancer types, and biomarkers that can identify the tumors that would be most responsive to specific immunotherapies are an area of active investigation (74). Candidate biomarkers of efficacy including PD-L1 expression, mutational burden, neoantigens, tumor infiltrating lymphocytes, and radiographic characteristics are under active study (75–79). Whether these same biomarkers will also predict responses to the combination of radiotherapy and immunotherapy remains an open question that should be actively addressed in ongoing and planned clinical trials.

There is also a need for biomarkers that can predict the occurrence of severe toxicity after the combination of immunotherapy and radiotherapy (80). Immune stimulatory drugs can cause immune-related adverse events (IrAEs) including fatigue, rash, skin disorders, and GI issues (81). Several large cohort studies (e.g. NCT03984318) are seeking to discover the underlying mechanisms responsible for severe IrAEs and identify predictive biomarkers. Biomarker candidate for IrAE prediction currently under investigation include cytokines, immune-cell subsets, autoantibodies, human leukocyte antigen haplotype, and radiomic characterization (82). Other studies are investigating the reduction of immunotherapy-related side effects through the use immunosuppressive drugs such as rituximab (anti-CD20) and tocilizumab (anti-IL-6) (NCT04375228). Radiotherapy is associated with its own set of toxicities, but can also cause adverse events similar to IrAEs through non-tumor specific antigens released into the tissue microenvironment by irradiation, potentially priming auto-reactive T cells to attack normal tissue (83). Predictors of these and other adverse events related to the combination of immunotherapy and radiotherapy need further study.

## Conclusion

A growing body of preclinical and clinical evidence indicates a potential synergy between radiotherapy and immunotherapy, lending support for the combination of these two treatment approaches. Unanswered questions remain regarding the optimal sequencing of treatment, dose fractionation, and biomarkers of response and toxicity. Within genitourinary cancers, multiple clinical studies are ongoing with early indications of both promising as well as negative results, suggesting that specific details regarding the protocol by which treatment is delivered may impact the overall success of the approach. These efforts are exemplified by the SWOG/NRG 1806 phase 3 study testing the addition of atezolizumab to chemoradiation in muscle-invasive bladder cancer. Should these initial trials show promise, confirmatory trials may be necessary given increased FDA scrutiny of immunotherapy in light of recent voluntary withdrawal of drugs that received accelerated approval in bladder cancer (84). Continued research efforts are needed to fully evaluate and optimize this promising combination of radiotherapy and immunotherapy.

## Author Contributions

JU, EK, and DM acquired, analyzed, and interpreted the data. JU, EK, and DM drafted and revised the manuscript for intellectual content. All authors contributed to the article and approved the submitted version.

## Funding

This work was funded in part by the Cygnus Montanus Foundation founded by the Svanberg family and the Massachusetts General Hospital.

## Conflict of Interest

The authors declare that the research was conducted in the absence of any commercial or financial relationships that could be construed as a potential conflict of interest.
